# Factors associated with difficulty in crossing the culprit lesion of acute myocardial infarction

**DOI:** 10.1038/s41598-021-00832-3

**Published:** 2021-11-01

**Authors:** Shun Ishibashi, Kenichi Sakakura, Satoshi Asada, Yousuke Taniguchi, Hiroyuki Jinnouchi, Takunori Tsukui, Kei Yamamoto, Masaru Seguchi, Hiroshi Wada, Hideo Fujita

**Affiliations:** Division of Cardiovascular Medicine, Saitama Medical Center, Jichi Medical University, 1-847 Amanuma, Omiya, Saitama City, 330-8503 Japan

**Keywords:** Cardiology, Interventional cardiology

## Abstract

In percutaneous coronary intervention (PCI) to the culprit lesion of acute myocardial infarction (AMI), unsuccessful guidewire crossing causes immediate poor outcomes. It is important to determine the factors associated with unsuccessful guidewire crossing in AMI lesions. The purpose of this study was to find factors associated with difficulty in crossing the culprit lesion of AMI. We defined the difficult group when the guidewire used to cross the culprit lesion was a polymer jacket type guidewire or a stiff guidewire. We included 937 patients, and divided those into the non-difficult group (n = 876) and the difficult group (n = 61). Proximal reference diameter was significantly smaller in the difficult group than in the non-difficult group (*p* < 0.001), and degree of calcification was severer in the difficult group than in the non-difficult group (*p* < 0.001). In the multivariate stepwise logistic regression analysis, proximal reference diameter [odds ratio (OR) 0.313, 95% confidence interval (CI) 0.185–0.529, *p* < 0.001)], previous PCI (OR 3.065, 95% CI 1.612–5.830, *p* = 0.001), moderate-severe calcification (OR 4.322, 95% CI 2.354–7.935, *p* < 0.001), blunt type obstruction (OR 12.646, 95% CI 6.805–23.503, p < 0.001), and the presence of collateral to the culprit lesion (OR 2.110, 95% CI 1.145–3.888, *p* = 0.017) were significantly associated with difficulty in crossing the culprit lesion. In conclusion, previous PCI, calcification, blunt type obstruction, and the presence of collateral were associated with difficulty in crossing the culprit lesion, whereas proximal reference diameter was inversely associated with difficulty. Our study provides a reference to recognize the difficulty in crossing the culprit lesions of AMI for PCI operators, especially junior operators.

## Introduction

Percutaneous coronary intervention (PCI) to the culprit lesion of acute myocardial infarction (AMI) accounts for the most significant portion of the contemporary treatment of AMI^1–3^. The mortality and morbidity of AMI have considerably decreased following the development of primary PCI^4,5^. Among several procedures in PCI, the first step for the successful revascularization is to manipulate the coronary guidewire to cross the culprit lesion of AMI, which is generally not so difficult for non-chronic total occlusion (CTO) lesions^6^, partly because more than half of the occluded segment of AMI consists of ruptured non-calcified plaque and platelet-mediated thrombus^7^. In fact, the delay in door-to-balloon time was affected primarily by the time delay from diagnosis in the emergency department to catheterization laboratory transfer rather than a delay in catheterization laboratory arrival to balloon inflation^8^.

However, even in primary PCI, interventional cardiologists sometimes encounter difficult cases in which the conventional guidewire cannot cross the culprit lesion. If the guidewire cannot cross the culprit lesion of AMI, emergent PCI results in unsuccessful reperfusion, which may lead to serious mechanical complications and subsequent poor clinical outcomes^9,10^. Several groups reported the determinants of successful or unsuccessful guidewire crossing in the field of PCI to CTO lesions^11,12^, whereas there has been few literatures regarding the determinants of guidewire crossing in PCI to AMI lesions. Since unsuccessful guidewire crossing would have more serious impact on patient’s immediate outcomes in PCI to AMI lesions than PCI to CTO lesions, it is of utmost importance to determine the factors associated with unsuccessful guidewire crossing in AMI lesions. The purpose of this study was to find factors associated with difficulty in crossing the culprit lesion of AMI.

## Methods

### Study design

We identified consecutive AMI patients in our institution from January, 2015 to December, 2018. The inclusion criteria were (1) patients with AMI, and (2) patients who underwent PCI to the culprit lesion of AMI. The exclusion criteria were (1) patients who were treated by medical therapy alone, (2) patients who underwent coronary artery bypass graft surgery to the culprit lesion, and (3) patients who underwent PCI to the non-culprit lesion as well as the culprit lesion of AMI simultaneously. The final study population was divided into a difficult group and a non-difficult group according to the guidewire used to cross the culprit lesion. The difficult group was defined as the lesion in which a conventional floppy guidewire failed to cross and a polymer jacket type wire or a stiff guidewire with a tip load of 1.5 g or more was required to cross. The non-difficult group was defined as the lesion in which a conventional floppy guidewire successfully crossed. The primary interest of this study was to find factors associated with the difficult group using multivariate logistic regression analysis. This study was approved by the Institutional Review Board of the Saitama Medical Center, Jichi Medical University (S20-136), and written informed consent was waived by the institutional review board of the Saitama Medical Center, Jichi Medical University, because of the retrospective study design. All methods were performed in accordance with the relevant guidelines and regulations.

### PCI to the culprit of AMI

PCI procedures were performed on a biplane fluoroscopy system. The choice of PCI devices such as guidewire, balloon, thrombectomy device, rotational atherectomy device, and stent was left at the discretion of interventional cardiologists at our cardiology center. First, we selected a conventional floppy wire to cross the culprit lesion of AMI. Our catheter laboratory had the following conventional floppy wire during the study period: Sion blue (Asahi Intecc, Aichi, Japan), Sion (Asahi Intecc, Aichi, Japan), Runthrough NS Floppy (Terumo, Tokyo, Japan), and ETNA (FMD, Tokyo, Japan). If the floppy wire could not cross the culprit lesion, we used a microcatheter to enhance backup or used a polymer jacket type guidewire. During the study period, the following polymer jacket type guidewires were used for this purpose: Sion black (Asahi Intecc, Aichi, Japan) and XT-R (Asahi Intecc, Aichi, Japan). Although microcatheters were used in most cases of lesions required polymer jacket guidewires, Sion black was sometimes used without microcatheter. If the polymer jacket type guidewire could not cross the lesion, we used a stiff guidewire with a tip load of 1.5 g or more. When we used a stiff guidewire, microcatheters were always used. During the study period, the following stiff guidewires were used for this purpose: Gaia First (Asahi Intecc, Aichi, Japan), Gaia Next 1 (Asahi Intecc, Aichi, Japan), ULTIMATE Bros 3 (Asahi Intecc, Aichi, Japan), and Conquest Pro 12 (Asahi Intecc, Japan). Our university hospital had many operators including junior operators. However, each PCI was strictly supervised by staff operators even in mid-night or holidays. Staff operators did not hesitate to take over procedures, when junior operators felt any difficulties in procedures such as the guidewire crossing the culprit lesion, the delivering device to the culprit lesion, etc. The decision to use a polymer jacket guidewire or a stiff guidewire was not made by junior operators, but by staff operators. Successful PCI was defined as angiographical residual diameter stenosis < 50% with decrease in minimum stenosis with TIMI flow grade ≥ 2^13^.

### Definition

Acute myocardial infarction (AMI) was defined based on universal definition of myocardial infarction^14,15^. Hypertension was defined as medical treatment for hypertension and/or a history of hypertension before admission^16^. Dyslipidemia was defined as a total cholesterol level ≥ 220 mg/dL or low-density lipoprotein cholesterol level ≥ 140 mg/dL or medical treatment for dyslipidemia or a history of dyslipidemia^16,17^. Diabetes mellitus was defined as a hemoglobin A1c level ≥ 6.5% (as NGSP value) or medical treatment for diabetes mellitus or a history of diabetes mellitus^16,17^. We calculated the estimated glomerular filtration rate (eGFR) from the serum creatinine level, age, weight, and gender using the following formula: eGFR = 194 × Cr-1.094 × age-0.287 (male), eGFR = 194 × Cr-1.094 × age-0.287 × 0.739 (female)^18^.

### Angiographical analysis

Quantitative coronary angiography (QCA) parameters were measured using a cardiovascular angiography analysis system (QAngio XA 7.3, MEDIS Imaging Systems, Leiden, Netherlands). QCA parameters such as lesion length and reference diameter were measured after the acquisition of reperfusion (after ballooning or thrombectomy) if the lesion was occluded. However, these QCA parameters might not be accurate especially in the measurement of distal reference diameter. We separately measured proximal reference diameter in all cases using the published method^19^. We did not use reference diameter or lesion lengths in the multivariate analysis, but used proximal reference diameter in the multivariate logistic regression analysis. The definition of lesion characteristics including lesion length, eccentricity, tortuosity, lesion angle, ostial lesion, bifurcation lesion has been previously described^20^. As known, bifurcation lesion is defined as the side branch requiring wire protection from the culprit lesion^20^. In an original ACC/AHA definition, tortuosity was defined as follows: stenoses distal to two bends were defined as moderately tortuous, and those distal to three or more bends were defined as excessive tortuosity^20^. However, since the above definition of tortuosity has poor objectivity, we additionally defined tortuosity as follows: “ ≤ 1” moderate to excessive bend (< 120°) was defined as mild tortuosity, “2” moderate to excessive bend (< 120°) was defined as moderate tortuosity, and “ ≥ 3” moderate to excessive bend (< 120°) was defined as excessive tortuosity^21^. Calcification was identified as readily apparent radiopacities within the vascular wall at the site of the stenosis, and was classified as none/mild, moderate (radiopacities noted only during the cardiac cycle before contrast injection), and severe (radiopacities noted without cardiac motion before contrast injection generally compromising both sides of the arterial lumen)^22^. The lesion morphology was classified into either blunt type or tapered type based on the definition of the J-CTO score^11^. The degree of collateral artery was classified as grade 0–3 based on the Rentrop classification, and the presence of collateral was defined as Rentrop grade ≥ 1^23^. Thrombus was classified based on the TIMI thrombus grade^24^.

### Statistical analysis

Data are shown as the mean ± SD or percentage. Categorical variables are presented as numbers (percentage), and were compared using the Pearson’s chi-square test. Continuous variables were compared between the groups using the unpaired Student’s test. Univariate and multivariate logistic regression analyses were performed to find the association between clinical variables and difficulty in crossing the culprit lesion of AMI. In this model, the difficult group was used as the dependent variable. In the multivariate stepwise logistic regression model, the selection of independent variables was derived from the results of univariate logistic regression analysis (*p* < 0.05 in univariate analysis). However, variables with missing values were not included in the model. Furthermore, when there are ≥ 2 similar variables, only one variable was entered into the multivariable model to avoid multi-collinearity. The multivariate logistic regression analysis with likelihood ratio statistical criteria using backward elimination method was performed. The odds ratio (OR) and the 95% confidence interval (CI) were also calculated. A P value < 0.05 was considered statistically significant. We analyzed all data by IBM SPSS statistics version 24 (Chicago, Illinois, USA).

## Results

**Figure 1:**
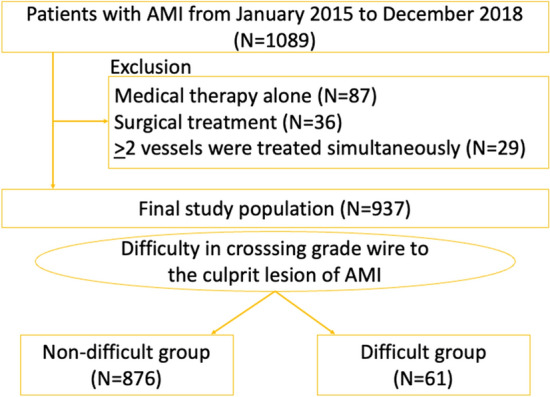
The study flow-chart. Abbreviations: AMI = acute myocardial infarction.

During the study period, we had 1089 AMI patients in our medical center. We excluded 152 patients according to the exclusion criteria. Our final study population was 937 patients, and was divided into the difficult group (n = 61) and the conventional group (n = 876). The study flowchart is shown in Fig. 1. The used guidewire in the difficult group were Sion black (n = 36), XT-R (n = 16), Gaia First (n = 3), Gaia Next 1 (n = 2), Ultimate Bros 3 (n = 3), and Conquest Pro 12 (n = 1), whereas the used guidewire in the non-difficult group were Sion blue (n = 664), Sion (n = 210), Runthrough NS floppy (n = 1), and ETNA (n = 1). There were no unsuccessful PCI cases in the final study population. There were 46 patients who changed their approach site from radial arteries to femoral arteries. Of 46 patients, 18 patients were due to radial artery spasm or narrowing, 17 patients were due to severe meandering of peripheral arteries such as brachiocephalic artery, and 11 patients were due to insufficient back-up support.

The comparison of clinical, lesion and procedural characteristics is shown in Table 1. Proximal reference diameter was significantly smaller in the difficult group than in the non-difficult group (2.43 ± 0.65 mm vs. 2.79 ± 0.65 mm, *p* < 0.001). Degree of calcification was severer in the difficult group than in the non-difficult group (*p* < 0.001). In the lesion morphology, the prevalence of blunt type was greater in the difficult group than in the non-difficult group (*p* < 0.001). Collateral to the culprit lesion was more common in the difficult group than in the non-difficult group (*p* = 0.001). There were no patients who required bi-directional approach (i.e. antegrade approach and retrograde approach) to complete PCI to the culprit lesion of AMI, but there were 5 patients who required contralateral injection to identify collateral arteries.

**Table 1:** Comparison of patient’s characteristics between the non-difficult group and the difficult group.

	All n = 937	Non-difficult group n = 876	Difficult group n = 61	*p*-value
Age, years	70.2 ± 12.5	70.0 ± 12.6	73.8 ± 9.7	0.027
Male, n (%)	711 (75.9)	669 (76.4)	42 (68.9)	0.185
**Physical examination**				
Height (cm)	161.7 ± 10.2 (n = 934)	161.9 ± 10.2 (n = 873)	158.9 ± 9.9	0.021
Body weight (kg)	63.1 ± 13.6 (n = 934)	63.3 ± 13.6 (n = 873)	60.5 ± 13.2	0.080
Systolic blood pressure at admission (mmHg)	140.3 ± 32.8 (n = 932)	140.0 ± 32.6 (n = 871)	144.7 ± 35.3	0.283
Diastolic blood pressure at admission (mmHg)	80.5 ± 20.5 (n = 931)	80.4 ± 20.3 (n = 870)	82.0 ± 22.6	0.566
Heart rate at admission (beat per minute)	82.0 ± 22.3 (n = 933)	81.4 ± 21.5 (n = 872)	89.3 ± 31.0	0.123
**Underlying disease**				
Hypertension, n (%)	762/936 (81.4)	708/875 (80.9)	54 (88.5)	0.140
Diabetes mellitus, n (%)	417/931 (44.8)	384/871 (44.1)	33/60 (55.0)	0.100
Dyslipidemia, n (%)	583/932 (62.6)	539/871 (61.9)	44 (72.1)	0.110
Hemodialysis, n (%)	89 (9.5)	79 (9.0)	10 (16.4)	0.057
History of previous PCI, n (%)	209 (22.3)	187 (21.3)	22 (36.1)	0.008
History of previous CABG, n (%)	38 (4.1)	34 (3.9)	4 (6.6)	0.306
History of previous MI, n (%)	148 (15.8)	140 (16.0)	8 (13.1)	0.553
Current smoker, n (%)	291/920 (31.6)	277/860 (32.2)	14/60 (23.3)	0.153
**Medication before admission**				
Aspirin, n (%)	304/930 (32.7)	273/870 (31.4)	31/60 (51.7)	0.001
Thienopyridine, n (%)	181/930 (19.5)	161//870 (18.5)	20/60 (33.3)	0.005
Beta-blocker, n (%)	222/911 (24.4)	203/852 (23.8)	19/59 (32.2)	0.147
ACE-inhibitor, ARB, n (%)	370/912 (40.6)	344/853 (40.3)	26/59 (44.1)	0.572
Calcium channel blocker, n (%)	347/910 (38.1)	325/851 (38.2)	22/59 (37.3)	0.890
Statin, n (%)	327/917 (35.7)	299/858 (34.8)	28/59 (47.5)	0.050
Diuretic, n (%)	124/915 (13.6)	112/856 (13.1)	12/59 (20.3)	0.115
Hypoglycemic agents, n (%)	242/920 (26.3)	217/861 (25.2)	25/59 (42.4)	0.004
Insulin, n (%)	63/922 (6.8)	57/863 (6.6)	6/59 (10.2)	0.294
**Laboratory data at admission**				
Serum creatinine (mg/dl)	1.66 ± 2.31	1.61 ± 2.24	2.30 ± 3.16	0.419
Estimated GFR (ml/min/1.73m^2^)	60.9 ± 31.8	61.0 ± 29.6	60.3 ± 54.7	0.242
Hemoglobin (g/dl)	13.2 ± 2.14	13.2 ± 2.09	12.5 ± 2.61	0.016
BNP (pg/ml)	420.3 ± 686.3 (n = 891)	394.4 ± 656.1 (n = 831)	778.3 ± 953.5 (n = 60)	< 0.001
**Killip class**				0.003
1 or 2	736 (78.5)	697 (79.6)	39 (63.9)	
3	99 (10.6)	85 (9.7)	14 (23.0)	
4	102 (10.9)	94 (10.7)	8 (13.1)	
Cardiac arrest at prehospital or ER, n (%)	55 (5.9)	48 (5.5)	7 (11.5)	0.054
Shock vital at prehospital or ER, n (%)	117 (12.5)	109 (12.4)	8 (13.1)	0.878
Admission within 24 h of onset	796/932 (85.4)	751/871 (86.2)	45 (73.8)	0.008
STEMI (vs NSTEMI)	563 (60.1)	533 (60.8)	30 (49.2)	0.072
**Culprit lesion**				0.456
Left main – left anterior descending artery, n (%)	477 (50.9)	443 (50.6)	34 (55.7)	
Right coronary artery, n (%)	311 (33.2)	296 (33.8)	15 (24.6)	
Left circumflex, n (%)	141 (15.0)	130 (14.8)	11 (18.0)	
Graft, n (%)	8 (0.9)	7 (0.8)	1 (1.6)	
**Number of narrowed coronary arteries**				0.031
1 vessel disease, n (%)	403 (43.0)	382 (43.6)	21 (34.4)	
2 vessel disease, n (%)	311 (33.2)	294 (33.6)	17 (27.9)	
3 vessel disease, n (%)	223 (23.8)	200 (22.8)	23 (37.7)	
Left main trunk lesion, n (%)	101 (10.8)	95 (10.8)	6 (9.8)	0.806
Left main trunk bifurcation lesion, n (%)	53 (5.7)	50 (5.7)	3 (4.9)	0.796
Ostia lesion, n (%)	77 (8.2)	69 (7.9)	8 (13.1)	0.150
Bifurcation lesion, n (%)	217 (23.2)	207 (23.6)	10 (16.4)	0.195
**Initial TIMI flow grade of culprit**				0.003
0	350 (37.4)	319 (36.4)	31 (50.8)	
1	88 (9.4)	79 (9.0)	9 (14.8)	
2	160 (17.1)	148 (16.9)	12 (19.7)	
3	339 (36.2)	330 (37.7)	9 (14.8)	
**TIMI Thrombus grade**				0.002
0	0	0	0	
1	479 (51.1)	458 (52.3)	21 (34.4)	
2	34 (3.6)	34 (3.9)	0	
3	50 (5.3)	49 (5.6)	1 (1.6)	
4	20 (2.1)	18 (2.1)	2 (3.3)	
5	354 (37.8)	317 (36.2)	37 (60.7)	
Lesion length (mm)	16.1 ± 10.2	16.0 ± 10.1	18.0 ± 11.7	0.279
Reference diameter (mm)	2.57 ± 0.75	2.59 ± 0.75	2.18 ± 0.65	< 0.001
Proximal reference diameter (mm)	2.72 ± 0.66	2.79 ± 0.65	2.43 ± 0.65	< 0.001
Tortuosity				0.481
Mild tortuosity, n (%)	815 (87.0)	765 (87.3)	50 (82.0)	
Moderate tortuosity, n (%)	87 (9.3)	79 (9.0)	8 (13.1)	
Excessive tortuosity, n (%)	35 (3.7)	32 (3.7)	3 (4.9)	
**Eccentricity**				0.088
Concentric, n (%)	871 (93.0)	811 (92.6)	60 (98.4)	
Eccentric, n (%)	66 (7.0)	65 (7.4)	1 (1.6)	
Calcification				< 0.001
None-mild, n (%)	709 (75.7)	680 (77.6)	29 (47.5)	
Moderate, n (%)	132 (14.1)	113 (12.9)	19 (31.1)	
Severe, n (%)	96 (10.2)	83 (9.5)	13 (21.3)	
**Type of obstruction site**				< 0.001
Blunt	171 (18.2)	131 (15.0)	40 (65.6)	
Tapered	766 (81.7)	745 (85.0)	21 (34.4)	
In-stent lesion	80 (8.5)	69 (7.9)	11 (18.0)	0.006
**The presence of collateral to the culprit lesion (Rentrop grade)**				0.001
0	701 (74.8)	668 (76.3)	33 (54.1)	
1	165 (17.6)	148 (16.9)	17 (27.9)	
2	66 (7.0)	56 (6.4)	10 (16.4)	
3	5 (0.5)	4 (0.5)	1 (1.6)	
Occurrence of slow flow in PCI, n (%)	63 (6.7)	61 (7.0)	2 (3.3)	0.267
**Approach site**				0.070
Trans-radial coronary intervention, n (%)	565 (60.3)	536 (61.2)	29 (47.5)	
Trans-brachial coronary intervention, n (%)	32 (3.4)	28 (3.2)	4 (6.6)	
Trans-femoral coronary intervention, n (%)	340 (36.3)	312 (35.6)	28 (45.9)	
**PCI procedure**				0.009
Plain old balloon angioplasty, n (%)	54 (5.8)	46 (5.3)	8 (13.1)	
Aspiration only, n (%)	6 (0.6)	6 (0.7)	0	
Drug coating balloon angioplasty, n (%)	42 (4.5)	37 (4.2)	5 (8.2)	
Bare metal stent, n (%)	23 (2.5)	22 (2.5)	1 (1.6)	
Drug eluting stent, n (%)	803 (85.7)	758 (86.5)	45 (73.8)	
POBA and aspiration, n (%)	6 (0.6)	4 (0.5)	2 (3.3)	
Other, n (%)	3 (0.3)	3 (0.3)	0	
**Size of guiding catheter (Fr)**				< 0.001
6	584 (62.4)	563 (64.3)	21 (34.4)	
7	343 (36.6)	307 (35.0)	36 (59.0)	
8	10 (1.1)	6 (0.7)	4 (6.6)	
**Number of guiding catheters**				< 0.001
1	868 (92.6)	822 (93.8)	46 (75.4)	
2	61 (6.5)	49 (5.6)	12 (19.7)	
≥ 3	8 (0.9)	5 (0.6)	3 (4.9)	
**Number of guide wires**				< 0.001
1	506 (54.0)	506 (57.8)	0	
2	315 (33.6)	287 (32.8)	28 (45.9)	
≥ 3	116 (12.4)	83 (9.5)	33 (54.1)	
**Number of stents**				0.002
0	121 (12.9)	105 (12.0)	16 (26.2)	
1	710 (75.8)	676 (77.2)	34 (55.7)	
2	98 (10.5)	88 (10.0)	10 (16.4)	
≥ 3	8 (0.9)	7 (0.8)	1 (1.6)	
**Number of balloons**				< 0.001
0	29 (3.1)	29 (3.3)	0	
1	409 (43.6)	392 (44.7)	17 (27.9)	
2	350 (37.4)	328 (37.4)	22 (36.1)	
≥ 3	149 (15.9)	127 (14.5)	22 (36.1)	
Use of guide-extension-catheter, n (%)	94 (10.0)	81 (9.2)	13 (21.3)	0.002
Use of microcatheter, n (%)	279 (29.8)	222 (25.3)	57 (93.4)	< 0.001
Use of aspiration catheter, n (%)	152 (16.2)	146 (16.7)	6 (9.8)	0.162
Use of rotational coronary atherectomy, n (%)	42 (4.5)	37 (4.2)	5 (8.2)	0.147
IVUS guide, n (%)	907 (96.8)	848 (96.8)	59 (96.7)	0.972
OCT/OFDI guide, n (%)	19 (2.0)	19 (2.2)	0	0.245
Temporary pacemaker, n (%)	67 (7.2)	63 (7.2)	4 (6.6)	0.852
Intra-aortic balloon pumping support, n (%)	97 (10.4)	90 (10.3)	7 (11.5)	0.766
V-A ECMO, n (%)	28 (3.0)	26 (3.0)	2 (3.3)	0.890
Amount of contrast media (ml)	126.6 ± 48.7	125.5 ± 47.8	142.8 ± 58.1	0.010
Fluoroscopy time (minutes)	24.7 ± 14.1	23.4 ± 12.5	43.0 ± 21.9	< 0.001

Data are expressed as the mean ± SD or number (percentage). A Student’s t test was used for normally distributed continuous variables, a Mann–Whitney U test was used for abnormally distributed continuous variables, and a chi-square test was used for categorical variables.

*PCI* indicates percutaneous coronary intervention, *CABG* coronary artery bypass grafting, *MI* myocardial infarction, *ACE* Angiotensin converting enzyme, *ARB* Angiotensin II receptor blocker, *GFR* glemerular filtration rate, *BNP* brain natriuretic peptide, *ER* emergency room, *TIMI* Thrombolysis in myocardial infarction, *IVUS* intravascular ultrasound, *OCT/OFDI* optical coherence tomography/optical frequency domain imaging, *V-A ECMO* veno-arterial extra-corporeal membranous oxygenation.

The univariate logistic regression model to find the factors associated with the difficulty in crossing the culprit lesion is shown in Table 2. Complex lesion characteristics such as small proximal reference diameter, calcification, blunt type obstruction, and the presence of collateral were associated with difficulty in crossing the culprit lesion. Furthermore, some procedural characteristics such as size of guiding catheter, number of guidewires, and use of microcatheter were also associated with the difficulty in crossing the culprit lesion.

**Table 2:** Univariate logistic regression analysis to find association with the difficulty in crossing the culprit lesion.

Independent variables	Odds ratio	95% confidence interval	*P* value
**Patient characteristics**			
Age	1.027	1.004–1.051	0.020
male sex	0.684	0.389–1.202	0.187
**Underlying disease**			
Height (per 10 cm incremental)	0.812	0.660–0.999	0.049
Body weight (per 10 kg incremental)	0.851	0.698–1.037	0.110
Systolic blood pressure (mmHg) (per 10 mmHg incremental)	1.049	0.969–1.135	0.236
Diastolic blood pressure (mmHg) (per 10 mmHg incremental)	1.037	0.915–1.174	0.571
Heart rate (beat per minute) (per 10 bpm incremental)	1.143	1.028–1.271	0.014
**Underlying disease**			
Hypertension	1.820	0.813–4.071	0.145
Diabetes mellitus	1.550	0.916–2.623	0.102
Dyslipidemia	1.594	0.896–2.836	0.113
Hemodialysis	1.978	0.967–4.049	0.062
History of previous MI	0.794	0.369–1.705	0.554
History of previous PCI	2.078	1.203–3.592	0.009
History of previous CABG	1.738	0.596–5.068	0.311
Current smoker	0.641	0.346–1.185	0.156
**Medication before admission**			
Aspirin	2.338	1.381–3.956	0.002
Thienopyridine	2.202	1.253–3.868	0.006
Beta-blocker	1.519	0.860–2.681	0.150
ACE-inhibitor, ARB	1.166	0.685–1.984	0.572
Calcium channel blocker	0.962	0.558–1.660	0.890
Statin	1.689	0.994–2.869	0.053
Diuretic	1.696	0.873–3.296	0.119
Hypoglycemic agents	2.182	1.273–3.740	0.005
Insulin	1.601	0.660–3.882	0.298
**Laboratory data at admission**			
Serum creatinine (mg/dl)	1.102	1.011–1.200	0.027
Estimated GFR (ml/min/1.73m^2^)	0.999	0.991–1.008	0.871
Hemoglobin (g/dl)	0.865	0.769–0.974	0.016
BNP (pg/ml) (per 100 pg/ml incremental)	1.055	1.027–1.083	< 0.001
**Killip class**			
4 versus 1–3	1.256	0.579–2.722	0.564
Cardiac arrest at prehospital or ER	2.236	0.966–5.177	0.060
Shock vital at prehospital or ER	1.062	0.492–2.294	0.878
Admission within 24 h of onset	0.449	0.246–0.821	0.009
STEMI (vs. NSTEMI)	0.623	0.370–1.047	0.074
**Lesion characteristics**			
*Culprit lesion*			
Left main—left anterior descending artery versus others	1.231	0.730–2.075	0.436
**Number of narrowed coronary arteries**			
3 vessel disease versus 1 or 2 vessel disease	2.046	1.191–3.515	0.010
Left main trunk lesion	0.897	0.376–2.139	0.806
Left main trunk bifurcation lesion	0.854	0.259–2.823	0.796
Ostia lesion	1.765	0.807–3.863	0.155
Bifurcation lesion	0.634	0.316–1.270	0.199
**Initial TIMI flow grade of culprit**			
3 versus 0–2	0.286	0.139–0.589	0.001
**TIMI Thrombus grade**			
4–5 versus 0–3	2.863	1.668–4.913	< 0.001
Lesion length (mm) (per 10 mm incremental)	1.189	0.968–1.461	0.099
Reference diameter (mm)	0.387	0.252–0.595	< 0.001
Proximal reference diameter (mm)	0.392	0.249–0.618	< 0.001
**Tortuosity**			
Excessive tortuosity versus mild-moderate tortuosity	1.364	0.406–4.589	0.616
**Eccentricity**			
Eccentric versus concentric	0.208	0.028–1.525	0.122
**Calcification**			
Moderate-severe versus none-mild	3.828	2.260–6.485	< 0.001
**Type of obstruction site**			
Blunt versus tapered	10.832	6.188–18.961	< 0.001
In-stent lesion	2.573	1.281–5.168	0.008
**The presence of collateral to the culprit lesion (Rentrop grade)**			
1–3 versus 0	2.777	1.639–4.706	< 0.001
Occurrence of slow flow in PCI	0.453	0.108–1.898	0.279
**Procedural characteristics**			
*Approach site*			
Trans radial coronary intervention versus TFI or TBI	0.575	0.342–0.967	0.037
**PCI procedure**			
Drug eluting stent versus others	0.438	0.240–0.800	0.007
**Size of guiding catheter**			
≥ 7 versus 6Fr	3.426	1.985–5.914	< 0.001
**Number of guiding catheter**			
≥ 2 versus 1	4.964	2.606–9.456	< 0.001
**Number of stent**			
≥ 2 versus 0–1	1.787	0.900–3.551	0.097
**Number of balloon**			
≥ 3 versus 0–2	3.327	1.909–5.798	< 0.001
Use of extension guide catheter	2.958	1.382–5.113	0.003
Use of micro catheter	41.98	15.060–117.019	< 0.001
Use of aspiration catheter	0.545	0.231–1.291	0.168
Use of rotational coronary atherectomy	2.025	0.766–5.353	0.155
Temporary pacemaker	0.906	0.318–2.577	0.853
Intra-aortic balloon pumping support	1.132	0.500–2.563	0.766
V-A ECMO	1.108	0.257–4.783	0.890

The multivariate stepwise logistic regression analysis to find the factors associated with difficulty in crossing the culprit lesion is shown in Table 3. The initial stepwise model included the following variables: age, proximal reference diameter, previous PCI, triple vessels disease (vs. ≤ 2 vessel diseases), initial TIMI flow grade 3 of culprit (vs. grade 0–2), moderate-severe calcification (vs. none-mild), blunt type obstruction (vs. tapered type), collateral to the culprit lesion, in-stent lesion, and TIMI thrombus grade 4–5 (vs. grade 0–3). In the final model, proximal reference diameter (OR 0.313, 95% CI 0.185–0.529, *p* < 0.001), history of previous PCI (OR 3.065, 95% CI 1.612–5.830, *p* = 0.001), moderate-severe calcification (vs. none-mild: OR 4.322, 95% CI 2.354–7.935, *p* < 0.001), blunt type obstruction (OR 12.646, 95% CI 6.805–23.503, *p* < 0.001), and the presence of collateral to the culprit lesion (OR 2.110, 95% CI 1.145–3.888, *p* = 0.017) were significantly associated with difficulty in crossing the culprit lesion.

**Table 3:** Multivariate logistic regression analysis model to find association with the difficulty in crossing the culprit lesion.

Dependent variable : the difficulty in crossing the culprit lesion			
Independent variables	Odds ratio	95% confidence interval	*P* value
Proximal reference diameter	0.313	0.185–0.529	< 0.001
Previous PCI	3.065	1.612–5.830	0.001
Moderate-severe calcification (vs. none-mild)	4.322	2.354–7.935	< 0.001
Blunt type of obstruction site (vs. tapered)	12.646	6.805–23.503	< 0.001
The presence of collateral to the culprit lesion (Rentrop grade1–3)	2.110	1.145–3.888	0.017

The stepwise model includes the following variables: age, proximal reference, previous PCI, number of diseased vessels, initial TIMI flow grade of culprit, calcification, type of obstruction site, the presence of collateral to the culprit lesion, In-stent lesion, TIMI thrombus grade.

## Discussion

We included 937 patients who underwent PCI to the culprit lesion of AMI, and divided those into the conventional group (n = 876) and the difficult group (n = 61) according to the difficulty in crossing the culprit lesion of AMI. In other words, approximately 7% of AMI lesions required a polymer jacket type guidewire or a stiff guidewire to cross the culprit lesion. In the multivariate stepwise logistic regression analysis, history of previous PCI, moderate-severe calcification, blunt type obstruction, and the presence of collateral to the culprit lesion were significantly associated with difficulty in crossing the culprit lesion, whereas proximal reference diameter was inversely associated with difficulty in crossing the culprit lesion.

In the present study, proximal reference diameter was inversely associated with difficulty in crossing the culprit lesion. Thus, the small vessel diameter was a factor associated with difficulty in crossing the lesion. In the pathology of AMI, there are 3 major underlying diseases: plaque rupture, plaque erosion, and calcified nodule^7,25^. Of these 3 underlying diseases, plaque rupture is closely associated with positive remodeling, whereas other 2 underlying diseases are not^26^. Therefore, the small vessel diameter may indicate that the underlying disease of the difficult cases were not plaque rupture, but plaque erosion or calcified nodule^27^. Although there were no studies to directly compare the difficulty in crossing lesions among 3 underlying diseases, most interventional cardiologists might be more familiar with crossing the lesion associated with plaque rupture rather than plaque erosion or calcified nodule.

We should discuss why history of previous PCI was associated with difficulty in crossing the lesion. History of previous PCI means that AMI occurred even after the patient had some secondary prevention drugs such as statin or aspirin^28^. Chronic statin treatment might reduce soft plaque components such as necrotic core^29^, which might affect the difficulty in crossing the lesion. Furthermore, most patients with history of previous PCI had coronary stents before AMI onset. Therefore, the type of occlusion in patients with history of previous PCI might be different from that in patients without history of previous PCI. For example, in-stent failure (restenosis or thrombosis) should be only observed in patients with history of previous PCI. However, it is still unknown why AMI lesions in patients with history of previous PCI was difficult to cross.

We should also discuss why moderate-severe calcification was associated with the difficulty in crossing the culprit lesion. Although there were few reports showing that calcification was the factor of difficulty in crossing the culprit lesion of AMI, it is widely known that calcification is associated with the difficulty of PCI^21^. Severe calcification causes unsuccessful device delivery, stent under-expansion, and long-term stent failure^30,31^. Moreover, moderate-severe calcification suggests that the underlying disease of AMI was calcified nodule rather than plaque rupture. When the underlying disease of AMI was calcified nodule, it might be difficult to cross the lesions by using conventional guidewires^32^, and might require polymer jacket type guidewires.

Blunt type obstruction was significantly associated with the difficulty in crossing the culprit lesion in the present study. In PCI to CTO lesions, it is widely known that it is more difficult to cross the lesion in blunt type obstructions than in tapered type obstructions^33^. Our results confirmed that blunt type obstruction can be a maker of the difficulty in crossing the lesion even in PCI to AMI lesions. The presence of collateral to the culprit lesion was also associated with the difficulty in crossing the lesion. Developed collateral arteries imply that there had been a severe stenosis before the complete obstruction occurred at the onset of AMI^34^, which suggests that the plaque components were not occupied by fresh thrombus, but by organized thrombus^35^.

In this study, the prevalence of admission within 24 h from onset was significantly less in the difficult group than in the non-difficult group. In general, thrombotic lesions are expected to become organized over time^36^, which made wire crossing difficult. Although we did not include admission within 24 h from onset in the multivariate logistic regression analysis because of missing value, lapsed time from the onset might be an important factor that determined the difficulty in crossing the culprit lesion of AMI.

Clinical implications of the present study should be noted. Unlike PCI to CTO lesions, junior PCI operators sometimes perform PCI to the culprit lesion of AMI without senior operator’s backup^37^, partly because AMI occurs anytime irrespective of on-hours or off-hours. Although most PCI to AMI lesions may be successfully managed by junior PCI operators, some AMI lesions require polymer jacket type guidewires or stiff guidewires which junior PCI operators are unfamiliar with. Our results would help junior PCI operators to consult with senior operators about backup support. If the culprit lesion morphology is blunt type obstruction with severe calcification, it may be better for junior PCI operators to ask senior operator’s support before trying polymer jacket type guidewires or stiff guidewires. Once the pseudo-lumen was enlarged by immature manipulation, it is very difficult even for senior operators to select the compressed true-lumen.

### Study limitations

First, because this study was a single-center retrospective observational study, there is a risk of patient selection bias. Second, we defined the difficult group as the lesion in which a conventional floppy guidewire failed to cross and a polymer jacket wire or a stiff guidewire with a tip load of 1.5 g or more was required to cross, which may lack objectivity. Although it would be better to define the unsuccessful guidewire crossing as the difficult case, there were no unsuccessful cases during the study period, probably because all AMI cases were supported by staff operators in our catheter laboratory. Since there were no cases when operators used polymer jacket type guidewires or stiff guidewires before trying conventional guidewires and the decision to use those specific guidewires was made by experienced staff operators, it may be acceptable to mention that our difficult cases were not technically easy. Furthermore, since most of our PCI cases were completed by the combination of junior operators and staff operators, it was difficult to adjust operator’s experience as one of confounding factors in multivariate analysis. Third, although we comprehensively judged the culprit lesion of AMI by reviewing angiogram, electrocardiogram, and echocardiogram, there is a possibility that we might misunderstand the culprit lesion in multivessel disease. We might perform PCI to CTO lesions as the culprit of AMI in multivessel disease, because we could not compare emergent angiogram with previous angiogram in most cases.

## Conclusions

In PCI to the culprit lesion of AMI, history of previous PCI, moderate-severe calcification, blunt type obstruction, and the presence of collateral to the culprit lesion were significantly associated with difficulty in crossing the culprit lesion, whereas proximal reference diameter was inversely associated with difficulty in crossing the culprit lesion. Our study provides a reference to identify difficulty in crossing the culprit lesions of AMI for PCI operators, especially junior operators.

## Data Availability

All data are available from the corresponding author on reasonable request.
